# Induction of CCL8/MCP-2 by Mycobacteria through the Activation of TLR2/PI3K/Akt Signaling Pathway

**DOI:** 10.1371/journal.pone.0056815

**Published:** 2013-02-13

**Authors:** Haipeng Liu, Zhonghua Liu, Jianxia Chen, Ling Chen, Xin He, Ruijuan Zheng, Hong Yang, Peng Song, Dong Weng, Haili Hu, Lin Fan, Heping Xiao, Stefan H. E. Kaufmann, Joel Ernst, Baoxue Ge

**Affiliations:** 1 Shanghai TB Key Laboratory, Shanghai Pulmonary Hospital, Tongji University School of Medicine, Shanghai, China; 2 Department of Microbiology and Immunology, Tongji University School of Medicine, Shanghai, China; 3 Clinical Translational Research Center, Shanghai Pulmonary Hospital, Tongji University School of Medicine, Shanghai, China; 4 TB Department, Shanghai Pulmonary Hospital, Tongji University School of Medicine, Shanghai, China; 5 Department of Immunology, Max Planck Institute for Infection Biology, Berlin, Germany; 6 Division of Infectious Diseases, New York University School of Medicine, New York, New York, United States of America; University of California, Riverside, United States of America

## Abstract

Pleural tuberculosis (TB), together with lymphatic TB, constitutes more than half of all extrapulmonary cases. Pleural effusions (PEs) in TB are representative of lymphocytic PEs which are dominated by T cells. However, the mechanism underlying T lymphocytes homing and accumulation in PEs is still incompletely understood. Here we performed a comparative analysis of cytokine abundance in PEs from TB patients and non-TB patients by protein array analysis and observed that MCP-2/CCL8 is highly expressed in the TB-PEs as compared to peripheral blood. Meanwhile, we observed that CCR5, the primary receptor used by MCP-2/CCL8, is mostly expressed on pleural CD4^+^ T lymphocytes. Furthermore, we found that infection with either *Mycobacterium bovis* Bacillus Calmette-Guérin (BCG) or *Mycobacterium tuberculosis* H37Rv induced production of MCP-2/CCL8 at both transcriptional and protein level in Raw264.7 and THP-1 macrophage cells, mouse peritoneal macrophages as well as human PBMC monocyte-derived macrophages (MDMs). The induction of MCP-2/CCL8 by mycobacteria is dependent on the activation of TLR2/PI3K/Akt and p38 signaling pathway. We conclude that accumulation of MCP-2/CCL8 in TB-PEs may function as a biomarker for TB diagnosis.

## Introduction

Tuberculosis (TB) continues to prevail as a major cause of mortality around the world killing almost 1.5 million people annually. Despite implementation of control programs and the availability of effective drugs, some 14% of TB patients suffer from tuberculous pleuritis, which presents as an acute illness characterized by fever, cough, pleural chest pain and pleural effusions (PEs). TB-PEs occur in approximately 2–10% of TB patients, which may result from primary or reactivation TB [1], [2]. The development of PEs is often associated with the accumulation of fluid enriched in proteins and cells in the pleural space [3]. It has been well documented that TB and cancer represent the two most frequent causes of exudative PEs predominated by lymphocytes in pleural fluid; whereas PEs during acute infections, including empyema and parapneumonic effusions, are typically characterized by influx of neutrophils [4], [5]. It is well known that bacterial, host and environmental factors influence the development of TB [6]. TB-PEs are caused by severe delayed-type hypersensitivity (DTH) reactions to the rupture of the subpleural focus of *Mycobacterium tuberculosis* (*Mtb*) infection. An improved understanding of the immunopathogenesis of TB-PEs can accelerate development of novel immunodiagnostic tools and therapeutic interventions.

The T cell response is a critical component of protective immunity against *Mtb* [7]. During mycobacterial infections, a strong CD4^+^ type 1 helper (Th1)-like immune response with prominent interferon-gamma (IFN-γ) secretion is considered critical for the containment of *Mtb* by means of activation of infected macrophages by Th1-type cytokines. However, Th1 cells alone do not explain the resistance/susceptibility to *Mtb* infection and TB disease [8]. Increasing evidence suggests that several Th subsets, including Th17 cells [9], regulatory T cells[10], Th9 cells [11] and Th22 cells [12], are involved in the pathogenesis of TB-PEs. A healthy pleural fluid contains few, if any, T cells. However, in TB-PEs, T cells preponderate, which are sequestrated in this compartment from both systemic and pulmonary vasculature on the visceral pleural surface and from systemic vessels from the parietal pleural surface. Homing of T cells to sites of infection and inflammation is incompletely understood. 

Chemokines are proinflammatory cytokines of low molecular size, which orchestrate migration and activation of different leukocyte populations. On the basis of the number and arrangement of conserved cysteins, chemokines can be divided into four groups; CXC, CC, C, and CX3C. Both the CXC and CC families contain many members, whereas lymphotactin and fractalkine/neurotactin are at present the only known C and CX3C chemokines, respectively. It has been reported that multiple chemokines expressed in the TB-PEs, including CXC chemokines such as IFN-γ-inducible protein of 10-kD (IP-10/CXCL10), monokine induced by IFN-γ (MIG/CXCL9), IFN-inducible T-cell alpha chemoattractant (I-TAC/CXCL11), interleukin-8 (IL-8/CXCL8), as well as CC chemokiines such as macrophage inflammatory protein-1 alpha (MIP-1alpha/CCL3), regulated upon activation normal T lymphocyte expressed and secreted (RANTES/CCL5) and monocyte chemotactic protein 1 (MCP-1/CCL2) [13], [14], [15], [16], [17]. Here we performed a protein array analysis of cytokine abundance in the PEs from TB patients and non-TB patients, and firstly identified MCP-2/CCL8 as a significantly higher expressed chemokine in the TB-PEs. MCP-2/CCL8 is a proinflammatory chemokine, which is expressed in inflamed tissues by resident and infiltrated cells (primarily monocyte/macrophages) after paracrine stimulation from T-cells by IFNs and other proinflammatory cytokines, or through innate mechanisms upon contact with viral, bacterial and fungal agents [18], [19]. It is chemotactic for, and activates, different cell types, including granulocytes and mononuclear phagocytes, through various chemokine receptors, including chemokine receptors CCR1, CCR2B and CCR5 [20], [21], [22]. In this study, we describe the source and regulation of MCP-2 as well as the expression of CCR5, which is the primary receptor of MCP-2 on cells accumulated in TB-PEs. Our study provides first hints on the role of MCP-2/CCL8 in the pathogenesis of pleural TB.

## Results

### Elevated MCP-2/CCL8 expression in TB-PEs

**Figure 1:**
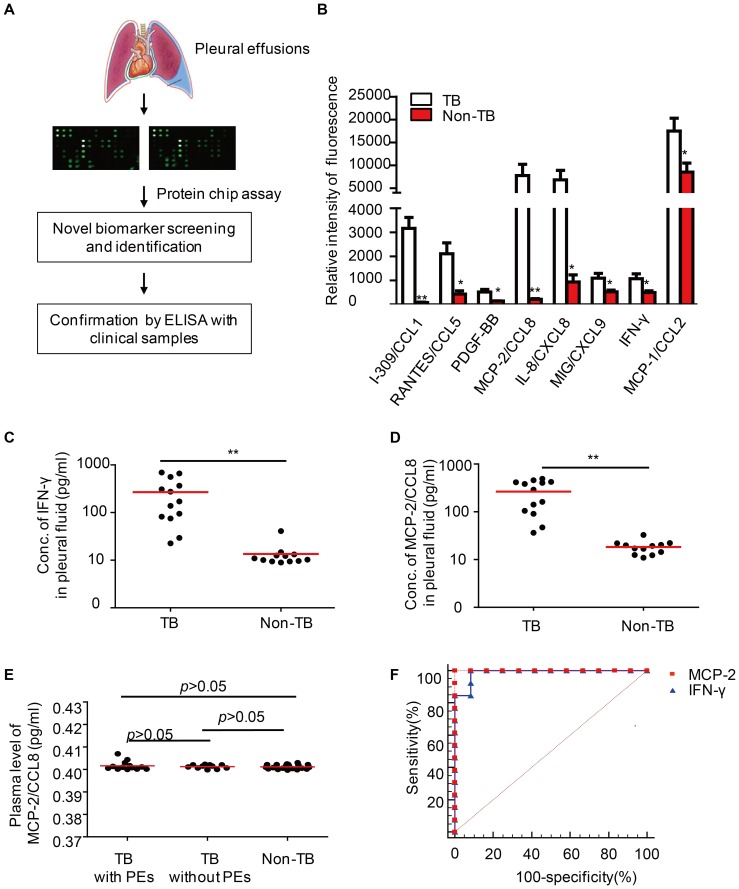
High MCP-2/CCL8 abundance in TB-PEs. (A) Diagram showed that PEs from both TB and non-TB patients were applied to protein chip assays to screen differentially expressed cytokines. Data were subsequently confirmed by ELISA. (B) Differentially expressed proteins in PEs from TB patients and non-TB patients. (C–D) ELISA of IFN-γ (C) or MCP-2/CCL8(D) in the PEs from TB or non-TB patients. (E) ELISA of MCP-2/CCL8 protein in the peripheral blood from TB patients with or without PEs and healthy donors. (F) Receiver operating characteristic curve showing the area under curve (AUC) of MCP-2/CCL8 and IFN-γ. **, *p*<0.001, *, *p*<0.05.

To understand the pathogenesis of pleural TB, we performed a protein array analysis to examine the expression of a total of 40 different cytokines between PEs from TB patients and those from non-TB patients, including cancer, pneumonia, and pulmonary arterial hypertension (PAH) **(** **Fig.1 A** **,** **Table 1** **)**. Concentrations of eight cytokines including I-309/CCL1, RANTES/CCL5, MCP-2/CCL8, IL-8/CXCL8, MIG/CXCL9, MCP-1/CCL2, IFN-γ and PDGF-BB, were significantly elevated in TB-PEs **(** **Fig.1 B** **)**. We then examined the expression of these cytokines by ELISA in PEs from 13 definite TB patients and 12 control patients in whom TB was definitely excluded. Pleural IFN-γ has been suggested for diagnosis of TB-PEs [23]. We consistently found higher expression of IFN-γ in TB-PEs than in non-TB-PEs **(** **Fig.1 C** **)**. We also firstly observed the presence of MCP-2/CCL8 in TB-PEs and identified it as a significant higher expressed chemokinein the TB-PEs as compared to malignant PEs **(** **Fig.1 D** **)**. However, MCP-2/CCL8 expression was low in the plasma of TB patients either with or without PEs, comparable to healthy donors **(** **Fig.1 E** **)**. These results suggest that MCP-2 accumulates in TB-PEs.

**Table 1:** Protein array analysis of cytokine abundance in PEs from TB patients and non-TB patients (Values stand for relative intensity of fluorescence).

Cytokines	TB (n = 4)	Non-TB (n = 4)	*p* value
	MEAN±SD	MEAN±SD	
I-309/CCL1	3165.176±914.1466	77.9323±36.2464	0.000516
RANTES/CCL5	2109.016±908.365	432.2511±274.1662	0.012303
PDGF-BB	515.3914±218.1624	140.5112±14.22901	0.013981
MCP-2/CCL8	7780.719±4906.762	209.8403±66.84131	0.021508
IL-8/CXCL8	6820.907±4172.937	929.9392±592.7471	0.031355
MIG/CXCL9	1094.831±404.285	524.6906±146.5638	0.037946
IFN-gamma	1069.531±411.7542	496.7534±139.3981	0.038788
MCP-1/CCL2	17483.35±5633.848	8505.757±3984.14	0.040548
IL-6	63399.42±10537.73	33269.22±25833.59	0.074091
IL-16	3318.953±1745.441	1352.52±793.1329	0.086079
IP-10/CXCL10	2788.125±638.7175	2083.75±386.1531	0.108032
IL-13	1155.284±510.6158	691.0185±214.8835	0.144738
IL-7	303.4743±43.92227	225.9394±84.65913	0.155096
TGF-beta 1	1258.264±579.7825	809.3893±276.8387	0.21181
TNF-alpha	665.3725±253.2047	470.3294±125.4151	0.216649
IL-15	1105.663±490.0397	747.0751±298.3578	0.25783
IL-12 p40	278.8993±244.9954	132.2073±20.76634	0.277818
IL-1alpha	789.1984±389.7232	501.7783±296.2249	0.284774
MIP-1beta/CCL4	6802.41±10969.83	411.3608±269.8152	0.288293
GM-CSF	646.3957±181.013	487.8681±213.2812	0.300297
MIP-1alpha/CCL3	1565.804±2724.014	145.5518±57.88581	0.33734
Eotaxin/CCL11	337.635±214.8732	207.8508±145.9365	0.356223
IL-1beta	347.0988±464.1977	118.85±25.54739	0.364057
MIP-1-delta/CCL15	4775.702±8371.438	738.1173±381.3618	0.372454
IL-11	209.9409±130.9967	126.0273±115.9656	0.374464
G-CSF	166.8412±7.549383	183.6784±38.35403	0.422074
TNF-beta	666.4918±197.8449	557.2063±168.022	0.432028
TIMP-2	4451.75±6831.38	8885.03±9518.633	0.477841
sTNF-RI	187.032±149.1322	146.2441±50.03695	0.622608
sTNF RII	6674.679±1196.531	6295.141±1381.866	0.692384
IL-4	54.49841±8.085172	65.69883±55.71437	0.704477
ICAM-1	1604.135±2342.114	2198.599±1883.139	0.706074
IL-2	570.7178±172.9008	512.4196±239.7293	0.706871
IL-3	437.4608±113.2187	461.0945±75.28243	0.739974
IL-10	1111.49±558.5364	975.8132±682.902	0.7688
IL-17	206.5566±75.81097	179.9974±158.1352	0.772196
IL-12 p70	104.0271±56.58202	109.7565±24.16727	0.858393
Eotaxin-2/CCL24	267.0551±235.2759	272.8658±228.7234	0.972896
IL-6 R	6293.626±2664.849	6248.495±1029.621	0.975819
M-CSF	111.9243±48.51104	110.8315±56.15117	0.977457

We next analyzed the diagnostic value of MCP-2/CCL8 in differential diagnoses of TB-PEs and PEs with the other etiologies. Using the receiver operating characteristic (ROC) curve, we noted that the area under curve (AUC) was 1.0 for MCP-2/CCL8 and 0.987 for IFN-γ in TB-PEs. With this cut-off value of 22.78 pg/ml for MCP-2/CCL8 and of 14.75 pg/ml for IFN-γ, sensitivity and specificity of MCP-2/CCL8 for TB-PEs were both 100%. Thus, MCP-2/CCL8 is a candidate marker for differential diagnosis of TB-PEs.

### Elevated percentage of CCR5-expressing CD4^+^ T cells in TB-PEs

**Figure 2:**
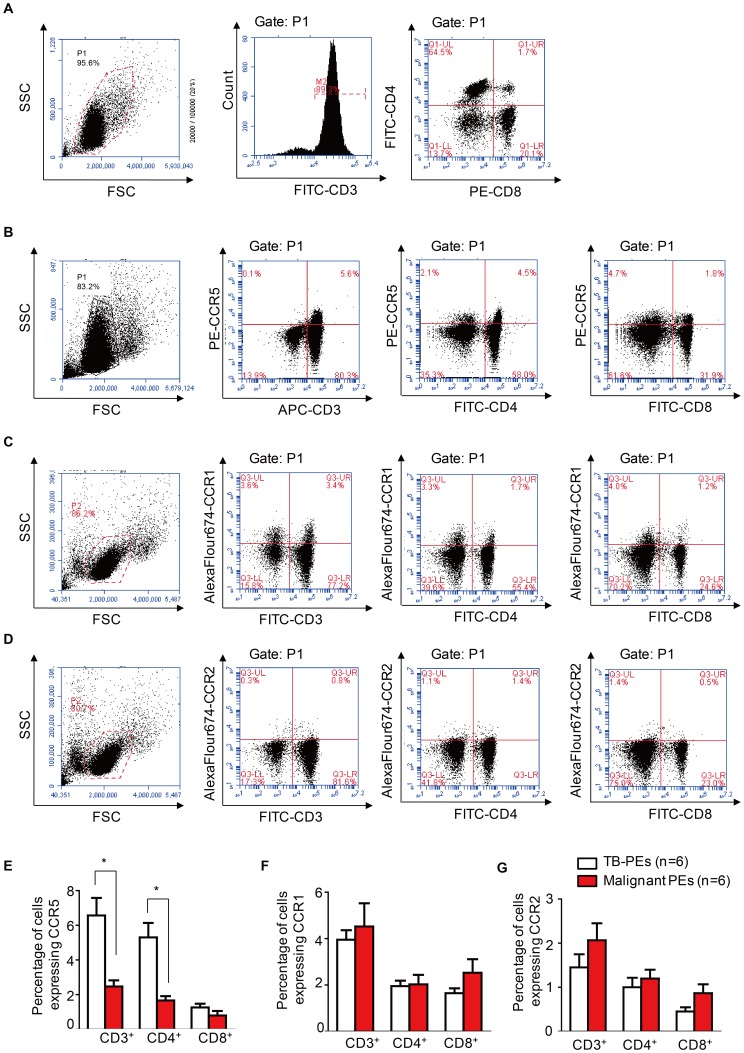
Higher percentage of CCR5-expressing CD4^+^ T cells in TB-PEs. (A) Representative FACS detection of CD3^+^, CD4^+^ and CD8^+^ cells in PEs from TB patients. (B–D) Representative FACS detection of the expression of CCR5(B), CCR1 (C) or CCR2 (D) on CD3^+^, CD4^+^ and CD8^+^ cells from TB-PEs. (E–G) Graph shows the comparison of the percentage of CCR5 (E), CCR1 (F) or CCR2 (G) expressing CD3^+^,CD4^+^ and CD8^+^ cells between TB-PEs and malignant PEs (n = 6). All the fluorescent antibodies are purchased from ebioscience (San Diego, CA). The fluorescent antibodies are used according to the manual.*, *p*<0.05

MCP-2/CCL8 is a proinflammatory chemokine that activates different cell types through various chemokine receptors, including CCR5. CCR5 has a promiscuous ligand-binding repertoire and is the primary receptor used by MCP-2/CCL8 on activated T cells [21]. Consistent with previous findings, TB-PEs were rich in lymphocytes, especially T cells (68.8%±26.7%, n = 6) **(** **Fig. 2A** **)**. Both CD4^+^ and CD8^+^ T cells were found in TB-PEs, with CD4^+^ T cells being dominant (49.6%±19.2% vs 19.6%±12.8%, n = 6) **(** **Fig. 2A** **)**. CCR5 was highly expressed on CD3^+^T lymphocytes but rarely on CD3^−^ lymphocytes in TB-PEs **(** **Fig. 2B** **)**, and both CD4^+^ and CD8^+^ T cells expressed CCR5 **(** **Fig. 2B** **)**. The percentages of CCR5-expressing CD3^+^ cells and CD4^+^ T cells in TB-PEs were significantly higher than those in malignant PEs (6.57%±1.02% vs 2.47%±0.36%, n = 6, p<0.05; 5.30%±0.84% vs 1.67%±0.25%, n = 6, p<0.05). Whereas expression of CCR5 on CD8^+^ T cells from TB-PEs and malignant PEs did not differ significantly (1.27%±0.21% vs 0.80%±0.26%, n = 6, p>0.05) **(** **Fig. 2E** **)**. Meanwhile, we detected the expression of CCR1 and CCR2, which are reported to be functional receptor of MCP-2/CCL8 [22], on T cells. We observed that CCR1 was expressed on both CD3^+^ and CD3^−^ lymphocytes **(** **Fig. 2C** **)**. In addition, the expression of CCR1 on CD3^+^, CD4^+^ and CD8^+^ T cells from TB-PEs was not significantly different from those from malignant PEs **(** **Fig. 2C, F** **)**. While there were very few expression of CCR2 on lymphocytes from either TB-PEs or malignant PEs **(** **Fig. 2D** **)**. Also the expression of CCR2 on different subsets of cells from TB-PEs is equivalent to those from malignant PEs. These results suggest that MCP-2/CCL8 recruits CD4^+^ T lymphocytes to TB-PEs through activating its primary receptor CCR5 instead of CCR1 or CCR2.

### Mycobacteria -induced MCP-2/CCL8 expression in macrophages

**Figure 3:**
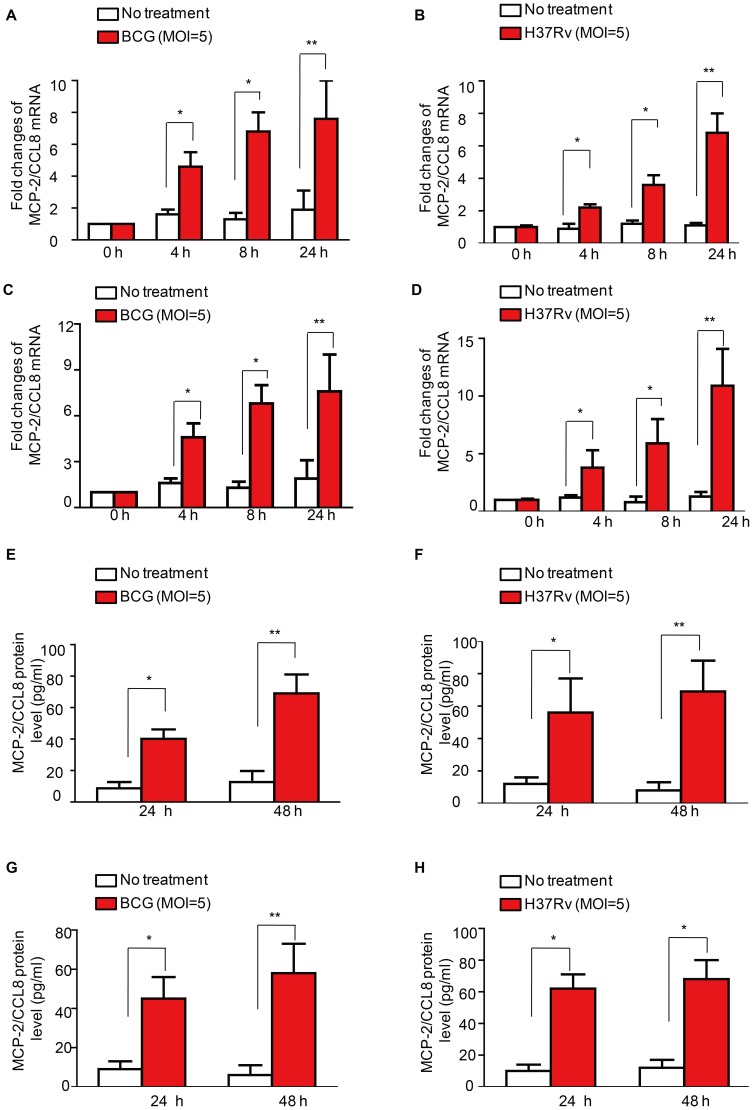
Mycobacteria-induced MCP-2/CCL8 expression in macrophages. (A–B) Real-time PCR detection of the production of *mcp-2/ccl8* mRNA in murine macrophage cell line Raw264.7 infected with either *M. bovis* BCG (A) or *M. tuberculosis* H37Rv (B) at MOI 5 for indicated times; (C–D) Real-time PCR detection of the production of *mcp-2/ccl8* mRNA in primary peritoneal macrophages treated with either *M. bovis* BCG (C) or *M. tuberculosis* H37Rv (D) at MOI 5 for indicated times; (E–F) ELISA of MCP-2/CCL8 protein in THP-1, a human acute monocytic leukemia cell line, infected with either *M. bovis* BCG (E) or *M. tuberculosis* H37Rv (F) at MOI 5 for indicated times. (G–H) ELISA of MCP-2/CCL8 protein in human PBMC monocyte-derived macrophages (MDM) infected with either *M. bovis* BCG (G) or *M. tuberculosis* H37Rv (H) at MOI 5 for indicated times. Data shown are representative of at least three independent experiments. **, *p*<0.001, *, *p*<0.05.

Macrophages are the primary target of the causative agent of TB, *Mtb* [24], [25], [26], and they have been found in TB-PEs [27]. To test whether Mtb infected macrophages are the origin of the elevated MCP-2/CCL8 in TB-PEs, we infected Raw264.7 cells, a murine macrophage cell line, with either *M. bovis* BCG or *M. tuberculosis* H37Rv at MOI 5. The mRNA level of *mcp-2/ccl8* in these mycobacteria-infected macrophage cells were analyzed by real-time RT-PCR **(** **Fig. 3A–B** **)**. BCG or H37Rv infection induced *mcp-2/ccl8* mRNA at different time points **(** **Fig. 3A–B** **)**. We also investigated whether *M. tuberculosis* H37Rv induced the synthesis of other elevated cytokines including *MCP-1/CCL2*, *RANTES/CCL5*, *MIG/CXCL9, I-309/CCL1* and *PDGF-BB* in Raw264.7 cells. *I-309/CCL1* is undetectable in Raw264.7 cells left untreated or treated with BCG. Other than a minor induction of *MIG/CXCL9* mRNA in Raw264.7 cells infected with BCG for 24 h, all other cytokines are not significantly induced **(Fig. S1)**. Therefore, macrophage may be the origin of MCP-2/CCL8 instead of MCP-1/CCL2, RANTES/CCL5, I-309/CCL1 and PDGF-BB. Next, we infected murine primary peritoneal macrophages with either BCG or H37Rv at MOI 5 and also observed an induction of the expression of *mcp-*2*/ccl8* mRNA at indicated time **(** **Fig. 3C–D** **)**. To make sure whether MCP-2/CCL8 could be induced by mycobacteria in human macrophages, we then infected phorbol-12-myristate-13-acetate (PMA)-differentiated THP1, a human acute monocytic leukemia cell line, with either BCG or H37Rv respectively at MOI 5 for 24 h and 48 h, and analyzed the production of MCP-2/CCL8 by ELISA. Both BCG and H37Rv infection markedly induced release of MCP-2/CCL8 protein from PMA-differentiated THP-1 macrophages **(** **Fig. 3E–F** **)**. Finally, we stimulated human PBMC monocyte-derived macrophage (MDM) with either BCG or H37Rv and then detected MCP-2/CCL8 released in the supernatant by ELISA. We observed that the MCP-2/CCL8 protein expression in MDM was also significantly induced by mycobacteria **(** **Fig. 3G–H** **)**. These results suggest that mycobacterial infection induces the expression of MCP-2/CCL8 at both transcriptional and protein levels in macrophages, which is of physiological relevance.

### Induction of MCP-2/CCL8 by mycobacteria through activation of p38 and PI3K/Akt

**Figure 4:**
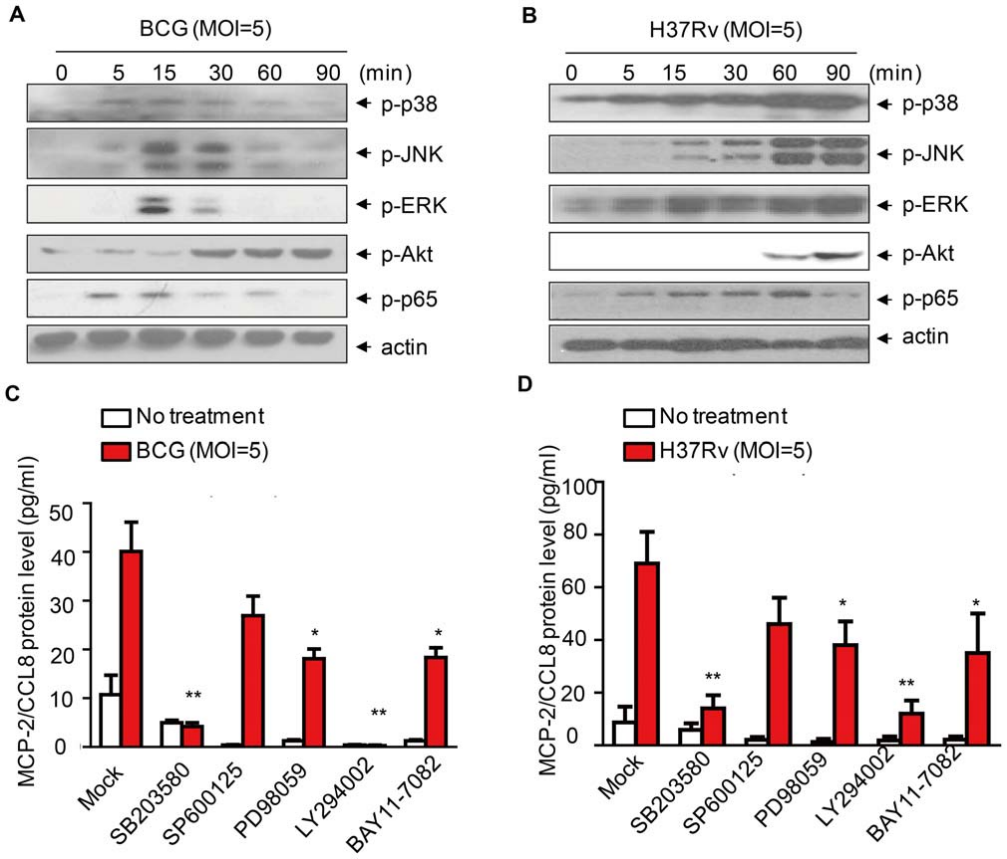
Mycobacteria-induced expression of MCP-2/CCL8 is regulated by p38 and PI3K. (A-B) Western blot of the signaling pathways including p38, JNK, ERK, Akt, NF-κB in mouse primary peritoneal macrophages infected with either *M. bovis* BCG (A) or *M. tuberculosis* H37Rv (B) at MOI 5 for indicated time. Data are representative of three independent experiments. (C) ELISA detection of MCP-2/CCL8 protein in THP-1 cells infected with *M. bovis* BCG at MOI 5 for 24 h in the presence of different kinase inhibitors, SB203580 (10 µM), SP600125 (10 µM), PD98059 (10 µM), LY294002 (10 µM), BAY11-7082 (10 µM). (D) ELISA detection of MCP-2/CCL8 protein in human PBMC monocyte-derived macrophages (MDM) infected with *M. tuberculosis* H37Rv at MOI 5 for 24 h in the presence of different kinase inhibitors as indicated in (C). **, *p*<0.001, *, *p*<0.05.

To elucidate the mechanisms underlying induction of MCP-2/CCL8 by mycobacteria in macrophages, we first analyzed the activation of MAPKs, including p38, ERK and JNK, the NF-κB signaling pathway as well as PI3K/Akt signaling pathway in BCG-infected murine primary peritoneal macrophages by immunoblot using anti-phosphor antibodies. We observed that BCG infection activates all these signaling pathways **(** **Fig. 4A** **)**. We also observed that H37Rv infection activates all these signaling pathways in a similar way **(** **Fig. 4B** **)**. To determine which signaling pathway is involved in the induction of MCP-2/CCL8 by mycobacteria, we infected THP-1 cells with BCG in the absence or presence of PI3K inhibitor (LY294002, 10 µM), ERK inhibitor (PD980259, 10 µM), JNK inhibitor (SP600125, 10 µM), p38 inhibitor (SB203580, 10 µM) or NF-κB inhibitor (BAY11-7082, 10 µM),and then detected the release of MCP-2/CCL8 in the supernatant by ELISA. Intriguingly, the induction of MCP-2/CCL8 was markedly inhibited by either SB203580 or LY294002. We also observed that SB203580 and LY294002 inhibited BCG-induced MCP-2/CCL8 production in a dose-dependent manner **(Fig. S2). Whereas, i**nhibition of ERK by PD98059 or inhibition of NF-κB by BAY11-7082 only partially inhibited the induction of MCP-2/CCL8 **(** **Fig. 4C** **)**. In addition, the inhibitory effect of these compounds on the induction of MCP-2/CCL8 in THP-1 cells are not due to the cytotoxic effect since the percentage of dead THP-1 cells treated with either DMSO or the inhibitors are equivalent **(Fig. S3A)**. We also replicated above experiment with human PBMC MDMs and we observed similar phenomena **(** **Fig. 4D** **and Fig. S3B)**. However, the induction of *mcp-2/ccl8* mRNA by BCG in Raw264.7 macrophages was significantly inhibited by SB203580, but not any other inhibitor tested, suggesting that activation of p38 is essential for the BCG-induced transcription of *mcp-2/ccl8* **(Fig. S4)**. Taken together, mycobacterial infection induces MCP-2/CCL8 expression in macrophages mainly through p38 and PI3K/Akt signaling pathways.

### TLR2 mediates mycobacteria-induced MCP-2/CCL8 production in macrophages

**Figure 5:**
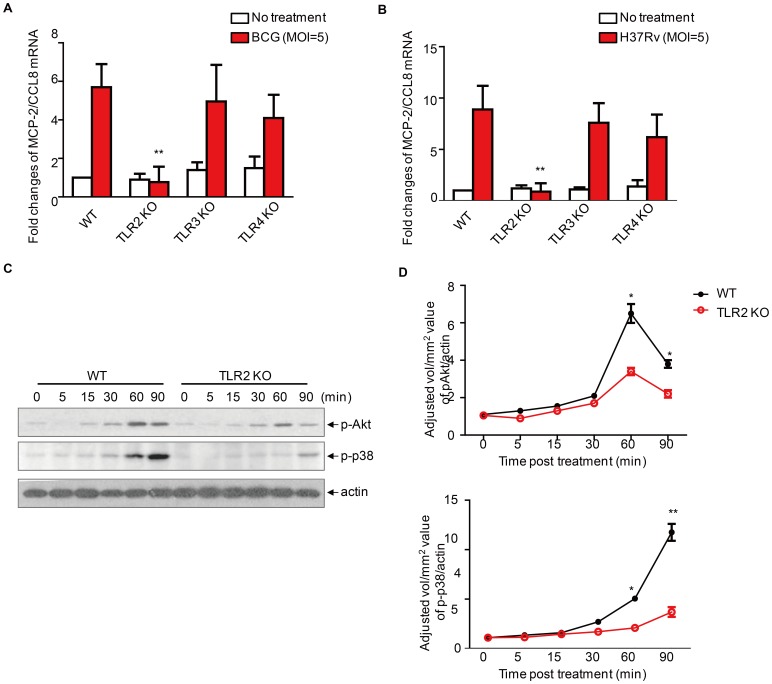
Mycobacteria-induced expression of MCP-2/CCL8 through TLR2. (A-B) Real-time PCR of *mcp-2/ccl8* mRNA in primary peritoneal macrophages from wildtype, TLR2^−/−^, TLR3^−/−^, or TLR4^−/−^ mice infected with either *M. bovis* BCG (A) or *M. tuberculosis* H37Rv (B) at MOI 5 for 24 h. (B) Western blot of phosphorylation of Akt and p38 in primary peritoneal macrophages from wildtype or TLR2 KO mice infected with *M. bovis* BCG at MOI 5 for indicated times. Data shown are representative of three independent experiments. (D) Densitometric analysis of three independent experiments as in (C). Data shown are the mean±sem of three independent experiments. **, *p*<0.001, *, *p*<0.05.

Pattern recognition receptors (PRRs) play an important role in the sensing of pathogens by means of pathogen associated molecular patterns (PAMPs) and the initiation of immune responses [28]. To interrogate which PRR is essential for mycobacteria-induced MCP-2/CCL8 production in macrophages, we compared mRNA levels of *mcp-2/ccl8* among macrophages from wild-type, TLR2^−/−^, TLR3^−/−^ or TLR4^−/−^ mice after infection with either BCG or H37Rv at an MOI 5 for 24 h. We observed that the *mcp-2/ccl8* mRNA level was significantly reduced in macrophages from TLR2^−/−^ mouse, but not in those from TLR3^−/−^ and TLR4^−/−^ mice **(** **Fig. 5A–B** **)**. Consistent with this observation, phosphorylation of Akt and p38 was markedly reduced in TLR2^−/−^ mice **(** **Fig. 5C–D** **)**. We therefore consider TLR2-mediated phosphorylation of Akt and p38 essential for induction of MCP-2/CCL8 by BCG in macrophages.

## Discussion

**Figure 6:**
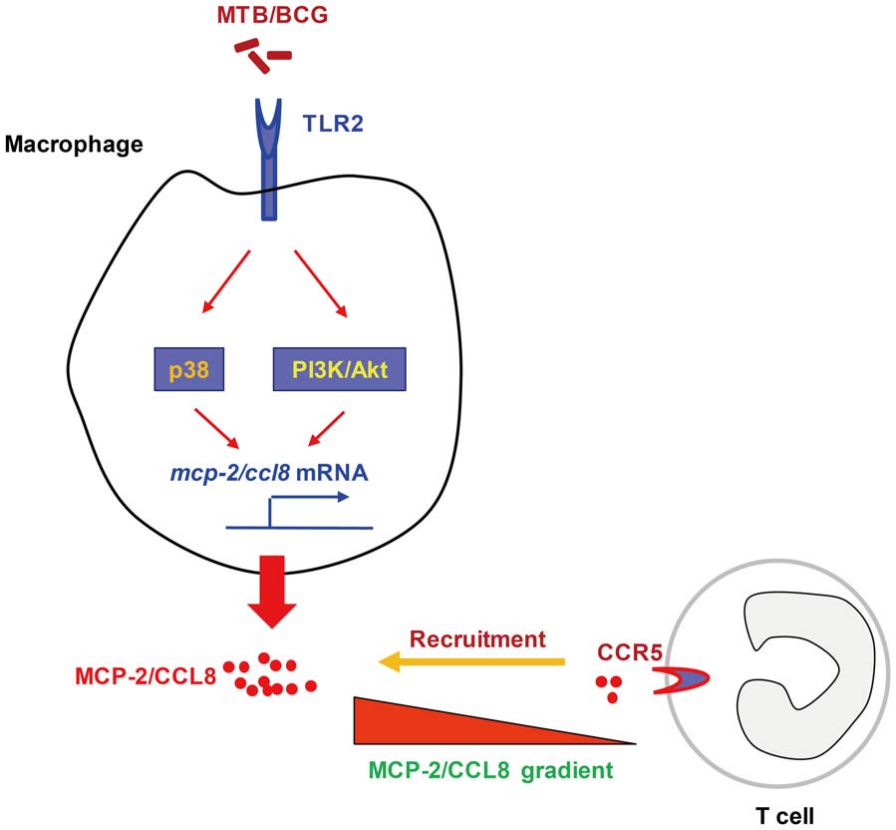
Diagram depicting recruitment of T lymphocytes expressing CCR5 in TB-PEs by mycobactera-induced expression of MCP-2/CCL8 in macrophages. Induction of MCP-2/CCL8 in macrophages by mycobacteria involves activation of TLR2/PI3K/Akt and p38 signaling. Highly expressed MCP-2/CCL8 in the TB-PEs may be responsible for the homing of CD4^+^ T lymphocytes into the infection site by activating the highly expressed CCR5, the primary receptor used by MCP-2/CCL8.

TB-related PEs are initiated once *Mtb* bacilli or antigen-derived monocytes, neutrophils, and subsequently T cells thereof reach the pleural space. Here we show that induction of MCP-2/CCL8 in macrophages by mycobacteria depends on activation of TLR2/PI3K/Akt and p38 signaling pathway. Furthermore, the highly expressed MCP-2/CCL8 in the TB-PEs likely induces influx of CD4^+^ T lymphocytes into TB-PEs by stimulated surface-expression of CCR5, the primary receptor for MCP-2/CCL8 **(** **Fig. 6** **)**.

*Mtb* antigens in pleural space elicit an intense immune response, initially by macrophages and neutrophils [29], [30] and followed by Th1 cells [31]. This series of events results in lymphocyte-rich exudative effusions. Accompanying the leukocyte infiltration, cytokines, chemokines, growth factors, and other soluble mediators are enriched in these TB-PEs, as demonstrated by our cytokine array results **(** **Table 1** **)**. It is widely accepted that these mediators orchestrate evolution of the disease, i.e., its resolution or its progression to fibrosis. Among the 40 cytokines detected and compared between TB and non-TB patients, 8 cytokines including I-309/CCL1, RANTES/CCL5, MCP-2/CCL8, IL-8/CXCL8, MIG/CXCL9, MCP-1/CCL2, IFN-γ and PDGF-BB were significantly higher expressed in TB-PEs. Intriguingly, 6 of the 8 cytokines were chemokines, emphasizing the important role of the chemokine network in TB-PEs. The Th1-type chemokines have been shown critical for the accumulation of CD4^+^ Th1 cells in TB-PEs which in turn are essential for *Mtb* containment[32]. Platelet-derived growth factor (PDGF)-BB is one of the five different isoforms of PDGF, which include A (PDGFA), B (PDGFB), C (PDGFC), D (PDGFD), and an AB heterodimer. Cellular responses are mediated by two different receptors, namely alpha (PDGFRA) and beta (PDGFRB). Production of PDGF-B by macrophages is considered central to pathogenesis of TB diseases involving chronic lung inflammation, which develops into fibrosis as a consequence of enhanced fibroblast proliferation and activation. Mycobacterial antigen-stimulated lymphocytes release IFN-γ, which in turn results in increased PDGF-B mRNA in alveolar macrophages [33]. The fibrogenic PDGF-BB decreases in serum after chemotherapy and is correlated with fibrosis [34]. These findings offer an explanation for the progression of chronic inflammation to fibrosis, as it occurs in the lungs of patients with untreated pulmonary TB [23], [35]. The high expression of PDGF-BB in TB-PEs shown here indicates its fundamental role in TB disease evolution and suggests its potential value for differential diagnosis of TB-PEs.

Mediators, including IFN-γ and adenosine deaminase (ADA) have been proposed as markers for differential diagnosis of PEs [36]. The estimated sum of sensitivity of IFN-γ was 89% (95% confidence interval, CI, 87–91%) and of specificity was 97% (CI, 96–98%). Apparently, these values are superior to published diagnostic accuracy of ADA [23], [35]. So far, alternative markers with similar or superior diagnostic power have not been identified. The responses of MCP-2/CCL8 to *Mtb*-specific antigens have been used for diagnosis of TB [37]. However, to our knowledge, the current study is the first to determine the discriminatory power of spontaneous MCP-2/CCL8 for TB-PEs versus PEs with the other etiologies. Intriguingly, we defined the cut-off value of 22.78 pg/ml for MCP-2/CCL8 and of 14.75 pg/ml for IFN-γ; sensitivity and specificity of MCP-2/CCL8 for TB-PEs were both 100%, and thus superior to diagnostic accuracy of IFN-γ with sensitivity of 100% but specificity of 91.7% for TB-PEs. We therefore suggest MCP-2/CCL8 as diagnostic parameter of TB-PEs. Due to the relatively small sample size and restricted diversity of PE etiologies, further prospective studies are required to validate the diagnostic value of MCP-2/CCL8 in larger cohorts with PEs of diverse etiologies.

Here we observed that the level of plasma MCP-2/CCL8 in TB patients is equivalent to those in healthy donors, distinct from the elevated plasma MCP-2/CCL8 levles in patients with sepsis [38], which suggested that the special constituents of MTB may be involved in the induction of MCP-2/CCL8. Though the biological activity of CCL8/MCP-2 in mice remains unclear, recent report showed that CCL8/MCP-2 in plasma increases with incidence of Graft-Versus-Host-Disease(GVHD) which is a complication of bone-marrow transplantation and the study suggests that MCP-2/CCL8 is associated with immune response within a living organism of mouse [39], [40]. We have done all experiment with both mouse and human cells and observed similar phenomena, suggesting that mouse MCP-2/CCL8 may have functional similarity with human MCP-2/CCL8.

Although induction of MCP-2/CCL8 in macrophages by mycobacteria at both transcriptional and protein levels has been previously reported [18], [41], underlying regulatory pathways have not yet been defined. Here we identify TLR2/PI3K/Akt and p38 signaling as essential pathway in mycobacteria-induced MCP-2/CCL8 production in macrophages. Our finding is consistent with a previous report that MCP-2/CCL8 secretion in rheumatoid arthritis synovial fibroblasts is mediated by TLR-2 [42]. Other studies reported that LPS induces expression of MCP-2/CCL8 in macrophages via MyD88-dependent signaling [43] and that poly(I:C) stimulates production of MCP-2/CCL8 in mononuclear cells[19]. We found that induction of MCP-2/CCL8 by mycobacteria only depended on TLR2 but not on TLR3 and TLR4. These differences are likely due to the different TLR-ligands studied and mycobacteria are rich in TLR2 agonists including lipoproteins such as LpdH, LprA, LprG, PhoS1, and glycolipids such as lipoarabinomannan (LAM), lipomannan (LM), phosphatidylinositol mannoside (PIM) and trehalose dimycolate (TDM) [44].

Chemokines and their receptors orchestrate leukocyte trafficking into and from lymphoid organs and tissues and hence are key regulators of immune responses. The TB-PEs are enriched for CD4^+^ Th1 cells critical for containment of *Mtb*. Understanding mechanisms involved in the selective recruitment of T lymphocytes to pathologic sites forms the basis for design of future intervention strategies. Here we demonstrate that T cells in TB-PEs highly surface-express CCR5. CCR5 has a promiscuous ligand-binding repertoire, including MIP1α/CCL3, MIP1β/CCL4 and RANTES/CCL5. It is the prime receptor used by MCP-2/CCL8 on activated T cells [21]. It has been reported that memory T cells obtained from the pleural fluid were shown to express a battery of homing receptors, including CCR5, which mediated adhesion of T cells from TB-PEs to activated human umbilical vein endothelial cells (HUVEC) expressing chemotactic ligands, which suggested the important role of CCR5 in the polarization of Th1 cells. [32] We conclude that highly expressed MCP-2/CCL8 in TB-PEs participates in homing of cells expressing CCR5. In our studies, CCR5 was expressed on both CD4^+^ T cells and CD8^+^ T cells. Yet, the percentage of CCR5-expressing CD4^+^ T cells, but not CD8^+^ T cells, in TB-PEs was significantly higher as compared to malignant PEs. Consequently, we assume that the high expression of MCP-2/CCL8 in TB-PEs recruited CD4^+^ T lymphocytes by activating its primary receptor CCR5. This notion is consistent with a previous report that high expression of MCP-2/CCL8 in mycoplasma-infected mice mediates recruitment and accumulation of CD4^+^ Th cells expressing CCR5 to the lung [45].

In conclusion, we defined soluble mediators in the PEs and identified MCP-2/CCL8 as valuable diagnostic marker for the differential diagnosis of PEs from TB and non-TB patients. Our data demonstrate that induction of MCP-2/CCL8 by mycobacteria depends on activation of TLR2/PI3K/Akt and p38 signaling pathway, and provide guidelines for design of novel diagnostic and therapeutic measures in TB based on the biomarker MCP-2/CCL8.

## Materials and Methods

### Cell Culture and Reagents

The murine macrophage cell line RAW264.7 (American Type Culture Collection, Manassas, VA) and THP-1 cells, a human acute monocytic leukemia cell line, were cultured in RPMI 1640, supplemented with 10% fetal bovine serum (FBS) and antibiotics (50 U/ml penicillin and 50 µg/ml streptomycin). THP-1 cells were pretreated with 50 ng/ml PMA (Phorbol-12-myristate-13-acetate) for 24 h to induce their differentiation before stimulation with *Mtb*.

RPMI 1640 medium, DMEM, FBS, penicillin, and streptomycin were purchased from Invitrogen (Shanghai, China). PI3K inhibitor (LY294002), ERK inhibitor (PD980259), JNK inhibitor (SP600125), p38 inhibitor (SB203580), nuclear factor-κB inhibitor (BAY11-7082) were obtained from Enzo Life Sciences (Farmingdale, NY), inhibitors are used at the concentration of 10 µM or otherwise indicated.. Abs against p-AKT, p-p38, p-ERK, p-JNK, p-p65 and actin were obtained from Cell Signaling Technology (Beverly, MA) and used for western bot with a dilution of 1∶1000. PE or FITC or APC labeled CD3, CD4, CD8 and CCR5 antibodies, AlexaFlour 647 labeled CCR1 and CCR2 as well as corresponding isotype control antibodies were all purchased from ebioscience (San Diego, CA). The fluorescent antibodies are used according to the manual. Normally, 5 µl fluorescent antibodies per test.

### Bacteria

*M. bovis* BCG and *M. tuberculosis* H37Rv were grown at 37°C with shaking in Middlebrook 7H9 broth (Becton Dickinson, Cockeysville, MD) with 0.05% Tween-80 and 10% oleic acid-albumin-dextrose-catalase(OADC) enrichment (Becton Dickinson, Sparks, MD). The bacterial suspension was centrifuged at 2,500 *g* for 10 min, and the pellet was suspended in RPMI 1640 plus 2 mM L-glutamine, 0.1 mM non-essential amino acids, 1 mM sodium pyruvate, and 20 mM sodium bicarbonate (Gibco BRL, Life Technologies, Grand Island, NY) (CRPMI). Bacteria were passed twice through a 27-gauge needle to disrupt clumps. Aliquots of 1 ml were stored at −80 °C for at least 1 day, and the inoculum was titrated by plating serial dilutions on Middlebrook 7H11 medium plus 10% OADC (both from Becton Dickinson Co., Sparks, MD).

### Animals and peritoneal macrophage isolation

TLR2^−/−^, TLR3^−/−^ and TLR4^−/−^ mouse mutants were originally from Professor S. Akira (Osaka University, Osaka, Japan) and were backcrossed eight times with C57BL/6. C57/BL6 mice were used as controls. All the mice were maintained in the Shanghai Laboratory Animal Center (SLAC, Shanghai, China), and used at 6 and 8 weeks of age. Peritoneal macrophages were isolated as described previously [46]. Briefly, mice were injected i.p. with 2 ml of 4% thioglycollate medium (Sigma-Aldrich). After 3 days, cells harvested by peritoneal lavage with cold PBS were allowed to adhere for 2 h and the RPMI 1640 medium was changed to remove non-adherent cells. The remaining adherent monolayer cells were used as primary peritoneal macrophages after at least 24 h. Before *M. bovis* BCG stimulation, cells were cultured in serum-free RPMI 1640 with antibiotics for 12 h to minimize the influence of FBS. All cells were cultured at 37°C in a humidified incubator with 5% CO_2_.

### Subjects

The study protocol was approved by the Medical School of Tongji University review boards for human studies, and informed consent was obtained from all subjects. Thirteen patients (8M/5F, 39.9±8.4 years) were proven to have TB-PE, as evidenced by growth of *Mtb* from pleural fluid or by diagnosis of granulomatous pleurisy on closed pleural biopsy specimen in the absence of any evidence of other granulomatous diseases. All TB-PE patients were anti-human immunodeficiency virus (HIV) antibody-negative and were recruited from the Shanghai Pulmonary Hospital. Following TB chemotherapy, the resolution of TB-PE and clinical symptoms was observed in all patients. At the time of sample collection, none of the patients had received any TB drug therapy, corticosteroids, or other non-steroid anti-inflammatory drugs. Twelve patients (6M/6F, 42.9±5.4 years) proven to have PEs with other etiologies, including cancer, pneumonia and PAH, were selected as comparators.

### Human peripheral blood mononuclear cells (PBMC) purification and monocytes-derived macrophages (MDM) differentiation

Human PBMC were purified with Lymphoprep solution (AXIS SCHIELD, Oslo, Norway) according to the manual. Briefly, 10 ml fresh human blood from healthy donors treated with heparin was collected and diluted with an equal volume of 0.9% (w/v) NaCl. Then 6 ml of diluted blood was carefully layered over 3 ml of Lymphoprep solution in a 15 ml conical centrifuge tube and were then centrifuged at 700 g for 30 min. The sharp band at the plasma/Lymphoprep interface which containing PBMC was recovered and washed with PBS. Then approximate 5×10^5^ PBMC were seeded in 96-well plate and left adhere for 1–2 h. The adhered cells are isolated monocytes. To allow freshly isolated monocytes to differentiate into MDM, the cells were cultured in the presence of 50 ng/ml human recombinant macrophage colony stimulating factor (M-CSF) for another six days by changing medium every two days. Normally, more than 95% of the differentiated cells are CD14^+^ (Data unshown). Then the cells were applied to infection by mycobacteria. All cells were cultured at 37°C in a humidified incubator with 5% CO_2_.

### PEs and serum isolation and enzyme-linked immunosorbent assay (ELISA)

PEs from TB patients and non-TB patients were collected in heparin-treated tubes through a standard thoracocentesis technique within 24 h after hospitalization. Ten ml of peripheral blood was drawn and centrifuged simultaneously. The supernatant collected above were analyzed for MCP-2/CCL8 and IFN-γ with ELISA according to the manufacturer's instructions (Raybiotech Inc.).

### Protein array analysis of cytokines

The cytokine concentrations in PEs were determined with RayBio Human Inflammation Antibody Array 3 (Cat# AAH-INF-G3-8) according to the manufacturer's instructions. Briefly, the glass chip was blocked and 100 µl PE samples were directly added to the subarray and incubated with array antibody supports, ensued with incubation with cocktail of biotinylated antibodies and subsequent incubation with labeled streptavidin. The glass chip was scanned with Axon GenePix (emission frequency 532 nm) to detect the fluorescent signals and the fluorescent intensity value was extracted with array analysis software and analyzed.

### RNA preparation, reverse transcription PCR, and quantitative real-time PCR

For reverse transcription PCR (RT-PCR), total RNA was isolated using TRIzol reagent (Invitrogen) following the manufacturer's instructions. Reverse transcription was done with the MMLV Reverse Transcription System (Invitrogen). Primers for quantitative real-time PCR (qRT-PCR) of the elevated cytokines in TB-PEs are listed in **Table 2**. Real-time PCR was run on Applied Biosystems 7300 Real-time PCR system with SYBR^®^ Green Real-Time PCR Master Mixes (TOYOBO). All PCR experiments were done in triplicate within each experiment, and experiments were replicated at least three times.

**Table 2:** Primers used for quantitative RT-PCR analysis.

Gene name	Gene ID	Primers	
I-309/CCL1	20290	Forward	GGCTGCCGTGTGGATACAG
		Reverse	AGGTGATTTTGAACCCACGTTT
MCP-1/CCL2	20296	Forward	TTAAAAACCTGGATCGGAACCAA
		Reverse	GCATTAGCTTCAGATTTACGGGT
RANTES/CCL5	20304	Forward	TTAAAAACCTGGATCGGAACCAA
		Reverse	GCATTAGCTTCAGATTTACGGGT
MCP-2/CCL8	20307	Forward	TCTACGCAGTGCTTCTTTGCC
		Reverse	AAGGGGGATCTTCAGCTTTAGTA
MIG/CXCL9	17329	Forward	GGAGTTCGAGGAACCCTAGTG
		Reverse	GGGATTTGTAGTGGATCGTGC
PDGF-BB	18591	Forward	CATCCGCTCCTTTGATGATCTT
		Reverse	GTGCTCGGGTCATGTTCAAGT
GAPDH	14433	Forward	AGGTCGGTGTGAACGGATTTG
		Reverse	GGGGTCGTTGATGGCAACA

### Flow cytometry analysis

PEs from patients were centrifuged at 1,000 g for 5 min. The cell pellets were resuspended with red blood cell (RBC) lysis buffer to remove RBCs and were then washed twice with PBS and fixed with 3.7% PFA. Then the cells were aliquoted and 5×10^5^ PE cells suspended in 100 µl staining buffer were mixed with 5 µl corresponding fluorescent antibodies and keep on ice for 20 min. Then the cells were washed with PBS twice. Flow cytometry analysis of cell surface markers included CD3, CD4, CD8, CCR1, CCR2 and CCR5. Samples were then run in the flow cytometer (BD Accuri C6) and subsequently analyzed with the BD CFlow Plus software.

### Macrophage infection and immunoblot analysis

Peritoneal macrophages were infected with either *M. bovis* BCG *M. tuberculosis* H37Rv at MOI 5 for indicated times. Subsequently, cells were lysed for 30 min on ice in lysis buffer, followed by centrifugation at 12,000 g at 4°C for 10 min. After centrifugation, supernatants were boiled in SDS loading buffer. Equivalent amounts of proteins were separated on SDS-PAGE under reducing conditions and were transferred to nitrocellulose membrane. ECL reagent (Thermo Scientific) was applied in an immunoblot analysis.

### Kinase inhibitors treatment and cytotoxicity assay

Cells were left treated with mock control or different kinase inhibitors with the concentration indicated 30 min before the infection of mycobacteria. The cytotoxic effect of these inhibitors was measured using the CytoTox96 Non-Radioactive Cytotoxicity Assay (Promega) according to the standard protocol, which is based on the detection of lactate dehydrogenase (LDH) released from damaged cells. The percent cytotoxicity was calculated as 100×Spontaneous/Maximum.

### Statistical Analysis

All data are expressed as mean ± SEM. Differences between groups were analyzed by the Student's t-test. Values of *p*<0.05 represented a statistically significant difference.

## Supporting Information

Figure S1 **Mycobacteria-induced cytokines expression in macrophages.** Real-time PCR detection of the mRNA of *MCP-1/CCL2* (A), *RANTES/CCL5* (B), *PDGF-BB* (C) and *MIG/CXCL9* in mouse macrophage cell line Raw264.7 cells infected with *M. tuberculosis* H37Rv at MOI 5 for indicated time. *, *p*<0.05.
(TIF)

Figure S2 **p38 inhibitor and PI3K inhibitor dose-dependently inhibit MCP-2/CCL8 production in macrophages.** ELISA detection of MCP-2/CCL8 protein in THP-1 cells infected with *M. bovis* BCG at MOI 5 for 24 h in the presence of p38 inhibitor (SB203580) and PI3K inhibitor (LY294002) with indicated concentration. *, *p*<0.05; **, *p*<0.001.
(TIF)

Figure S3 **Kinase inhibitors don't show significant cytotoxic effect on macrophages.** The cytotoxic effect of the different kinase inhibitors, SB203580 (10 µM), SP600125 (10 µM), PD98059 (10 µM), LY294002 (10 µM), BAY11-7082 (10 µM), on PMA-differentiated THP-1 cells (A) and PBMC monocyte-derived macrophages (MDMs) (B) was measured by detecting the release of lactate dehydrogenase (LDH) from damaged cells incubated with above inhibitors for 24 h.
(TIF)

Figure S4 **Activation of p38 is essential for mycobacteria-induced** ***mcp-2/ccl8*** **mRNA expression.** Real-time PCR detection of *mcp-2/ccl8* mRNA in murine macrophage cell line Raw264.7 cells infected with *M. bovis* BCG at MOI 5 for 24 h in the presence of kinase inhibitors, SB203580 (10 µM), SP600125 (10 µM), PD98059 (10 µM), LY294002 (10 µM), BAY11-7082 (10 µM). *, *p*<0.05.
(TIF)
