# Supplementary figures and images for: Induction of CCL8/MCP-2 by Mycobacteria through the Activation of TLR2/PI3K/Akt Signaling Pathway
Source: PLoS One. 2013 Feb 13;8(2):e56815. doi: 10.1371/journal.pone.0056815 (PMC3572057; doi:10.1371/journal.pone.0056815)

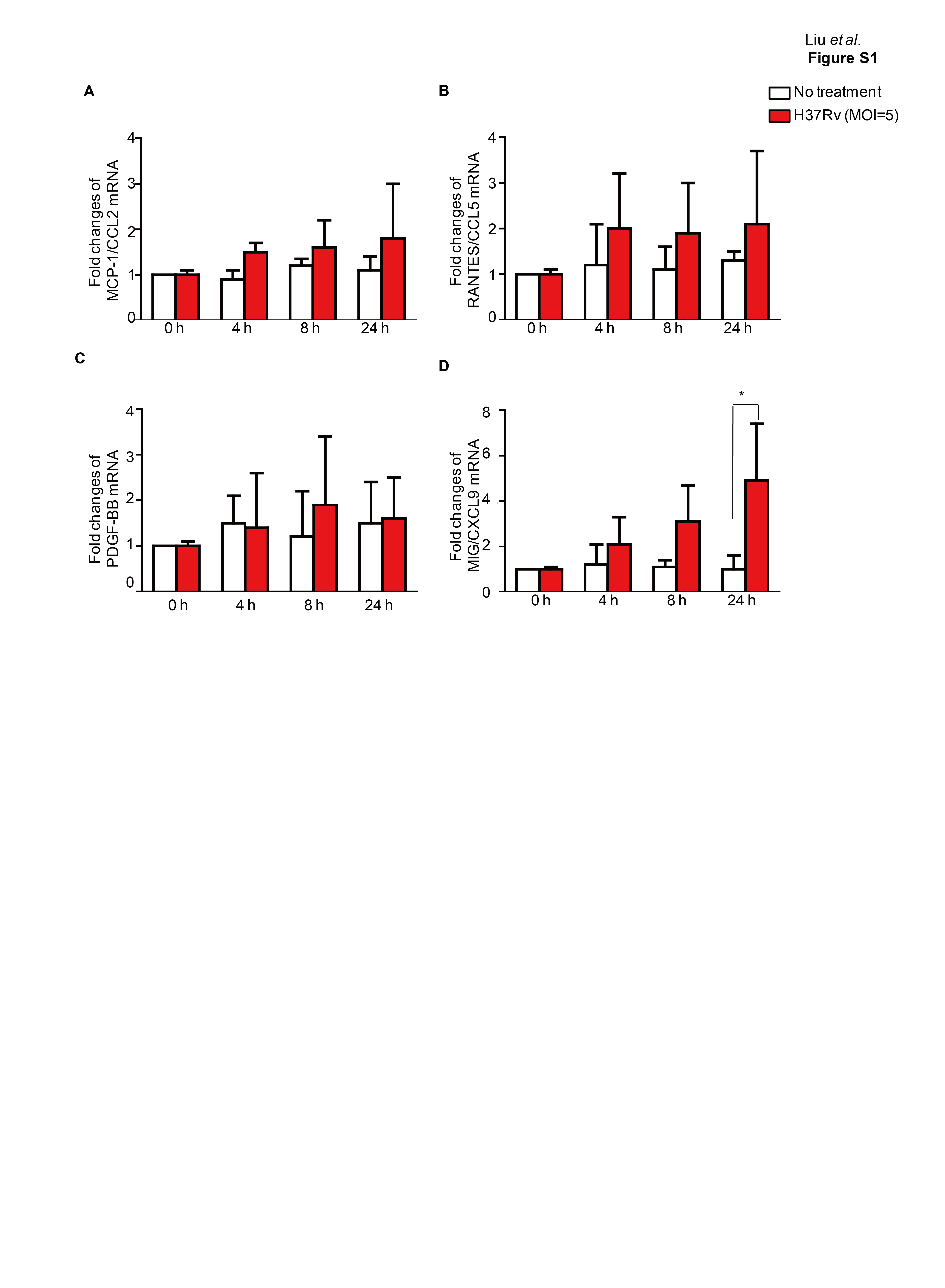

Supplement: Figure S1 — Mycobacteria-induced cytokines expression in macrophages. Real-time PCR detection of the mRNA of MCP-1/CCL2 (A), RANTES/CCL5 (B), PDGF-BB (C) and MIG/CXCL9 in mouse macrophage cell line Raw264.7 cells infected with M. tuberculosis H37Rv at MOI 5 for indicated time. *, p<0.05. (TIF) [file pone.0056815.s001.tif]
